# Sternoclavicular Septic Arthritis and Surgical Intervention: A Case Report

**DOI:** 10.7759/cureus.53002

**Published:** 2024-01-26

**Authors:** Abdullah Alnasser, Zeyad S Alamari, Taif M Almutairi, Hamid T Aljohani, Ahmed m Almulla

**Affiliations:** 1 Orthopaedic Surgery, Prince Sultan Military Medical City, Riyadh, SAU; 2 Orthopaedics, Prince Sultan Military Medical City, Riyadh, SAU; 3 College of Medicine, King Abdullah International Medical Research Center, King Saud bin Abdulaziz University for Health Sciences, Riyadh, SAU; 4 Orthopaedics and Traumatology, Prince Sultan Military Medical City, Riyadh, SAU

**Keywords:** conservative and surgical treatment, shoulder range of motion, empirical antibiotic therapy, medial clavicle resection, sternoclavicular joint (scj) septic arthritis

## Abstract

Management of septic arthritis is an area of controversy, especially in rare locations like the sternoclavicular joints. In this case report, we present a case of septic sternoclavicular joint, which was resistant to medical treatment and deteriorated during the treatment course. Although medical treatment has proven effective based on previous literature, some cases will still not benefit from it. In this case, our patient responded significantly to surgical treatment regarding upper limb function, faster infection eradication and rehabilitation, and shorter hospitalization and antibiotics duration.

## Introduction

Septic arthritis is a common and urgent condition in clinical practice with high morbidity and mortality [1]. Several pathogens cause septic arthritis, usually bacterial, such as *Staphylococcus aureus* and *Streptococcus *species, which account for approximately 90% of the cases [2]. Infrequently, fungi, parasites, viruses, or other atypical pathogens may be encountered [3]. Typically, septic arthritis affects a single major joint, such as the knee or hip [4]. Uncommonly, septic arthritis can be present in the sternoclavicular joint (SCJ) [1]. However, it is relatively rare in individuals without underlying medical conditions and accounts for less than 2% of the cases [1,4]. Several risk factors have been associated with an increased susceptibility to developing septic arthritis of the SCJ [5]. These risk factors include diabetes mellitus, rheumatoid arthritis, immunosuppression, intravenous drug use, traumatic events, and underlying arthropathies [5,6]. Septic arthritis of the SCJ has been recognized as a potentially life-threatening condition due to its close anatomical proximity to critical vascular structures in the chest [7]. Patients frequently report sudden onset of chest or shoulder pain, accompanied by or without fever [8]. Warmth and redness around the afflicted joint, as well as other signs of joint inflammation, may occur [9]. In addition, other cases may present with less common manifestations, such as abscess formation and subcutaneous emphysema [10]. Imaging modalities such as computed tomography (CT) and magnetic resonance imaging (MRI) are utilized for the evaluation of infection severity [6]. Joint aspiration or biopsy should be performed if an infection of the SCJ is suspected [8]. Controversy persists on the most effective way to treat SCJ infections [6,8,11]. In addition to long-term antibiotics, most authors believe that surgery is often necessary [8,11].

## Case presentation

**Figure 1:**
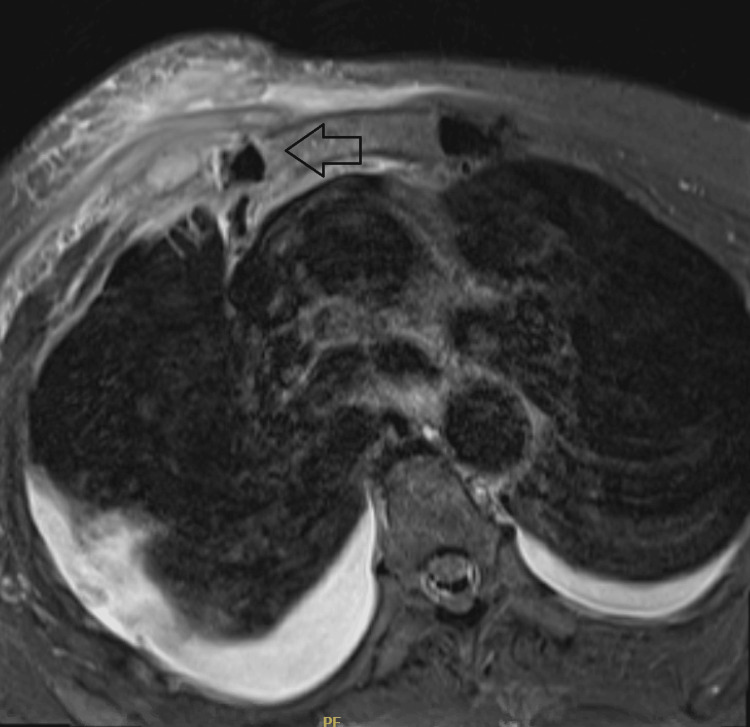
Preoperative right SCJ MRI showing large intra-articular joint effusion, with a collection deep to pectoralis major insertion and cutaneous reactions suggestive of an infective process (arrow pointing to findings). SCJ: sternoclaviclar joint

In this report, we present the case of a 70-year-old female who presented to our hospital with a three-day history of pain located in her right SCJ. Past medical history was significant for diabetes mellitus. She denied having experienced any recent trauma. However, she had a history of a positive urinary tract infection (UTI) a month prior to her presentation. The patient was afebrile and vitally stable. Local examination revealed only mild tenderness and swelling over the SCJ. Laboratory results revealed an elevated white blood cell count of 13.29x109, an erythrocyte sedimentation rate of 107, and a C-reactive protein level of 99. A CT without contrast showed soft tissue changes suggestive of a collection measuring approximately 3.6 cm in craniocaudal dimension and likely extending into the right SCJ. There was no definite bone destruction. Septic arthritis of the right SCJ was suspected, and an MRI was recommended for further evaluation (Figure 1). The MRI revealed right SCJ joint septic arthritis with phlegmon/collection anterior to the joint, in addition to another predominant inflammatory phlegmon deep to the sternal insertion of the right pectoralis major muscle. A diagnosis of SCJ septic arthritis was made, and the patient underwent surgical irrigation and debridement through a direct anterior clavicle approach. The pus drainage was measured to be 20 to 40 ml. Antibiotics were administered to the patient after a tissue culture was obtained. Cefazolin and vancomycin were administered intravenously for a total of 14 days, then clindamycin was continued orally for a total of 14 days.

**Figure 2:**
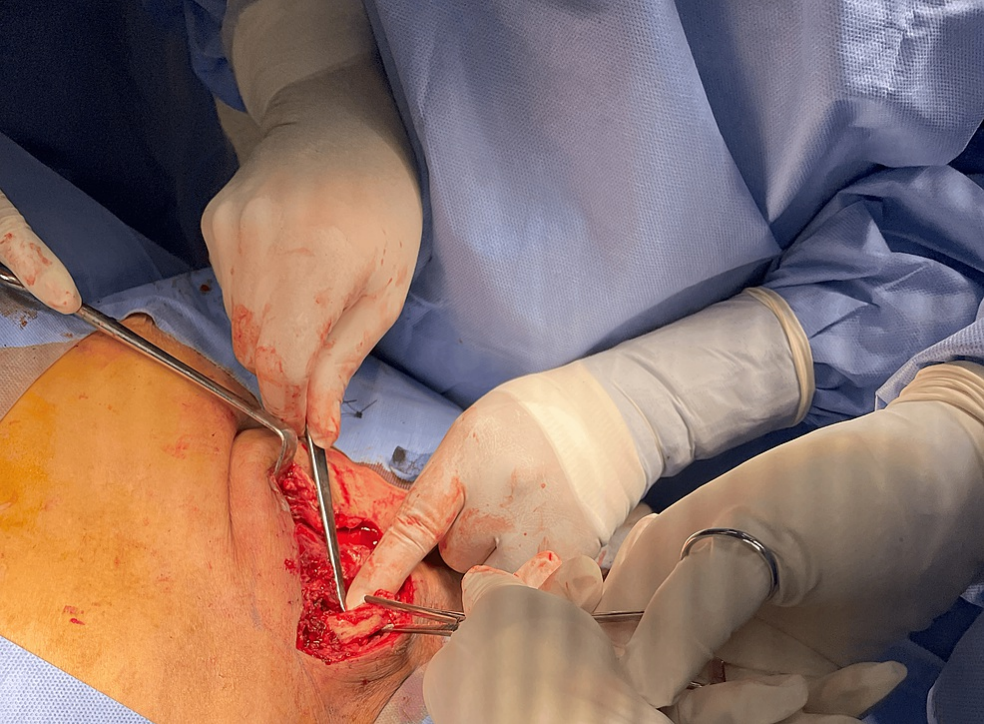
Intraoperative picture of medial clavicle length of resection as marked by Kocher forceps.

**Figure 3:**
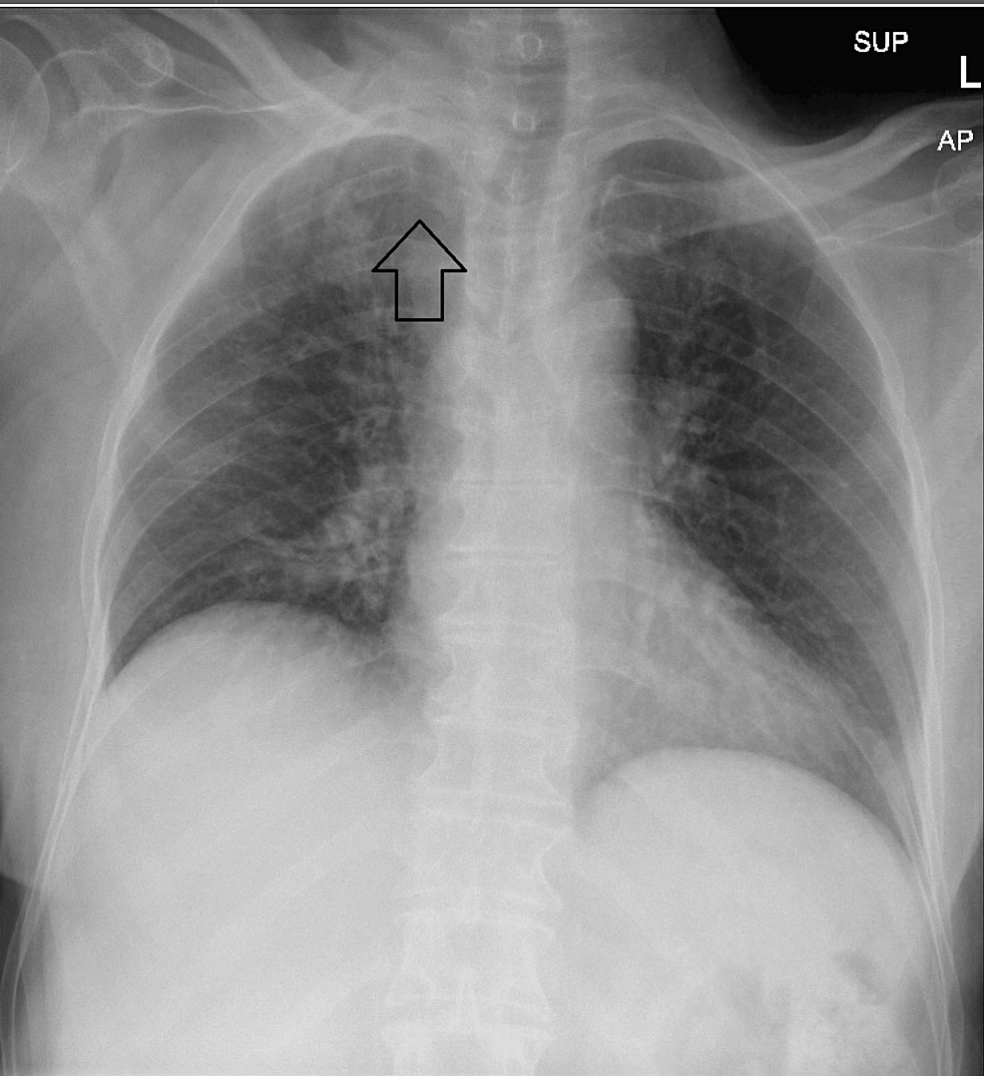
Postoperative X-ray showing the status post right medial clavicle resection

**Figure 4:**
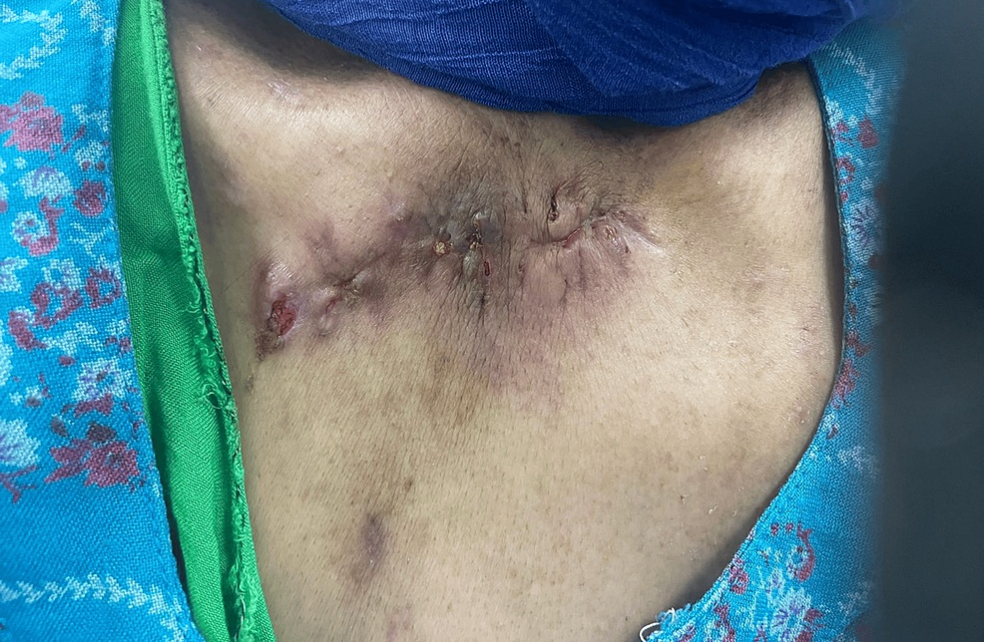
Two months postoperative surgical wound showing good healing.

The initial culture came positive for *Streptococcus agalactiae* (Group B) and biopsy findings were necrotic tissues with visible inflammatory cells confirming the diagnosis of septic arthritis. After completion of the antibiotics course, postoperatively on Day 1, a reoccurrence was evident clinically with a recurrence of symptoms, and recollection was observed on repeated images. The decision was made after patient counseling for surgical intervention in the form of medial clavicle resection and thorough irrigation and debridement. The same incision was opened, and a large amount of pus was evacuated. The incision was extended medially and laterally over the clavicle to reach the necrotic tissue. A large amount was evacuated from the anterior chest under the pectoralis muscle. The SCJ was dislocated, and a small amount of pus was found. The joint was clearly damaged and necrotic. The medial clavicle was excised until reaching healthy bone and sent for histopathology as shown in Figure 2. Following that, irrigation and debridement were carried out. Another course of intravenous antibiotics was prescribed. A postoperative X-ray indicated that the patient showed significant improvement since Day 1, regained her right upper limb function daily, and scored well based on the Disability of Arm, Shoulder, and Hand (DASH) questionnaire (Figure 3). Two months postoperatively, the patient’s wound healed, and she regained full range of motion of the right shoulder with no complaints (Figure 4).

## Discussion

Streptococci have been acknowledged as a common etiological factor in the development of septic arthritis, contributing to approximately 20% of all cases, being the second most common cause after *Staphylococcus aureus*, which accounts for 50-60% of cases [12]. Group B streptococcus (GBS), also known as *Streptococcus agalactiae*, has recently been recognized as a well-known cause of septic arthritis, accounting for 5-10% of all cases [12]. There are several risk factors identified in the literature that make patients more likely to be predisposed to GBS septic arthritis. In our case, both her advanced age (over 60 years of age) and gender (female) have been linked to the development of GBS [13]. Early identification and management are crucial in SCJ septic arthritis due to its close anatomical proximity to critical vascular structures [7] and the infection's tendency to spread beyond the joint due to the capsule's inability to distend [14]. As a result, patients are prone to experience serious complications such as emphysema, osteomyelitis, mastitis, and abscess formation [6]. Determining the optimal management strategy can be challenging in cases of SCJ septic arthritis. To this day, a consensus has yet to be reached regarding the most effective treatment modality, thus representing a topic of debate [6,8,11]. The fundamental principles of management include the eradication of infection, preservation of joint function, and reduction of pain [6]. Treatment options range from antibiotic therapy to radical surgery, including SCJ resection requiring muscle flap coverage [15]. Antibiotic therapy alone or in conjunction with isolated aspiration or needle lavage has been proposed, but there is insufficient evidence to support its efficacy [8]. Jang et al.'s study examined the outcomes of medical management in patients with* S. aureus* sternoclavicular septic arthritis [16]. Patients were enrolled in the study, and they were all managed medically or with limited surgery (incision-drainage and debridement) [16]. The average duration of the antibiotic course was 35 days [16]. The study found that all cases were successfully treated, with no recurrence or deterioration [16].

According to Jang et al., in selected patients without significant complications, medical treatment alone or in combination with limited surgery represents a successful management strategy [16]. On the other hand, Abu Arab et al. investigated the role of surgery in 14 patients with SCJ septic arthritis who had failed medical treatment with antibiotics [17]. All surgically treated patients had excellent outcomes with no restrictions in shoulder movements or recurrence of the infection [17]. In contrast to Jang et al. [16], the study recommended surgical intervention as the most effective treatment following the failure of the antibiotic trial [17]. Additionally, in a retrospective study, Song et al. evaluated minimally invasive interventions such as antibiotic administration, surgical drainage, and debridement in six patients [18]. The study reported a failure rate of 83% [18]. Reflecting on our case, we attempted medical management using simple irrigation and debridement in addition to IV antibiotics. Unfortunately, the infection persisted, necessitating a more aggressive intervention with medial clavicle resection. Our patient recovered well from the surgery with no complications and regained full joint function. Prompt intervention and infection eradication are essential for preventing prolonged hospitalization and serious complications resulting from chronic infection [6]. Additionally, reports of adverse outcomes in patients managed conservatively, including recurrence or persistence of infection, eventually leading to surgical therapy, have been documented [17,19]. Studies reporting the success of medical management were exclusive to *S. aureus* infection. A case report by Cydylo et al. described a case where the patient responded to oral trimethoprim-sulfamethoxazole; this case is the first to treat sternoclavicular septic arthritis with outpatient oral antibiotics. However, the patient refused aspiration and was treated empirically [20]. Furthermore, Jang et al.'s study was limited to *S. aureus* infections. In our case, the patient's culture was positive for GBS [16].

Many studies have suggested that medical management demonstrated success in achieving desired clinical outcomes in selective cases, such as patients presenting with a local infection with no complications. However, in situations where medical treatment fails, or serious complications occur, surgical intervention is a highly effective approach that can reduce the risk of complications and decrease morbidity and mortality rates.

## Conclusions

In this case, which showed rapid deterioration despite limited surgery and medical treatment, surgical intervention in the form of medial clavicle resection proved to be very effective in shortening patient hospitalization, enabling the patient to recover faster and resume daily activities. Although, based on previous literature, medical treatment has shown to be an effective model of treatment for sternoclavicular joint arthritis but still surgical intervention is a valid option for cases that are reluctant to receive medical treatment. Further research is needed to prove the effectiveness of medial clavicle resection in terms of hospitalization and the recovery of physical activity.
